# Dynamics of caste and early childbearing in India: a perspective of three decades

**DOI:** 10.1186/s12905-024-03077-0

**Published:** 2024-04-10

**Authors:** Manas Ranjan Pradhan, Sourav Mondal, Daisy Saikia, Prasanna Kumar Mudi

**Affiliations:** 1 Department of Fertility and Social Demography, International Institute for Population Sciences (IIPS), Govandi Station Road, Deonar, Mumbai, Maharashtra 400088 India; 2 International Institute for Population Sciences (IIPS), Govandi Station Road, Deonar, Mumbai, Maharashtra 400088 India

**Keywords:** Caste, Early childbearing, India, Three decades

## Abstract

**Background:**

Early childbearing disrupts girls’ otherwise healthy growth into adulthood and adversely affects their education, livelihood, and health. Individual, sociocultural, economic, environmental, and health service-related factors contribute to childbearing among young females. In India, caste affects health outcomes despite several affirmative policies aimed at improving the health and welfare of the backward castes/tribes. However, there is a dearth of empirical evidence about the impact of caste on early childbearing, more specifically, regarding the trajectory of inter-caste disparities in early childbearing.

**Method:**

This study used data from all five rounds of the National Family Health Survey (NFHS) in India to assess the association between caste and early childbearing over the last three decades. All women aged 20–24 [NFHS-1 (*n* = 17,218), NFHS-2 (*n* = 15,973), NFHS-3 (*n* = 22,807), NFHS-4 (*n* = 122,955) and NFHS-5 (*n* = 118,700)] were considered to create a pooled data set (*n* = 297,653) for analysis. Bivariate analysis and binary logistic regression were conducted using Stata (v17). ArcMap (v10.8) presented the caste-wise prevalence of early childbearing among the states and Union Territories (UTs).

**Results:**

Many women continue to have early childbearing despite a considerable reduction over the last three decades from 47% in 1992-93 to 15% in 2019-21. Compared to NFHS-1, the odds of early childbearing increased by 15% in NFHS-2 and, after that, declined by 42% in NFHS-3 and 64% in NFHS-4 and NFHS-5. The inter-caste disparity in early childbearing persists, albeit with a narrowing gap, with the Scheduled castes (SC) remaining the most vulnerable group. Adjusting the effects of socio-demographic and economic characteristics, SC women had significantly higher odds of early childbearing (OR = 1.07, CI = 1.04–1.11) than those from the General caste.

**Conclusion:**

To decrease early childbirth, a focus on adolescent marriage prevention and increasing contraceptive use among young SC women is necessary. Strengthening ongoing programs and policies targeting educational and economic empowerment of the socially weaker castes/tribes will help in reducing early childbearing. Efforts to prevent early childbearing will accelerate the achievement of the Sustainable Development Goals (SDGs)-especially those related to health, poverty, nutrition, education, and general wellbeing, in addition to protecting women’s reproductive rights.

## Background

Early childbearing, or pregnancy and delivery during adolescence, can disrupt girls’ otherwise healthy growth into adulthood and adversely affect their education, livelihood, and health [1]. Early childbearing harms the mother because complications during pregnancy and childbirth are the primary cause of death in girls between 15 and 19 [2]. It further affects neonatal morbidity and mortality and child development in various ways [3–5]. In 2021, an estimated 14% of adolescent girls and young women worldwide gave birth before the age of 18. The rates of early childbearing in India continue to be high [6]. Sociocultural, economic, and environmental factors i.e., coercive sexual relations, poverty, religion, early marriage, absence of affordable education, and non-use of contraceptives [7–9]; individual factors, i.e., excessive use of alcohol, substance abuse, educational status, low self-esteem, and inability to resist sexual temptation [8, 10, 11]; and health service related factors, i.e., cost of contraceptives, inadequate and unskilled health workers, long waiting time and lack of privacy at clinics, lack of comprehensive sexuality education, and non-friendly adolescent reproductive services [8, 12] contribute to early childbearing. Early marriage, school dropout, and early childbearing are frequently linked, and all three are influenced by poverty. Young women who quit school early are more likely to marry and have children sooner than those who stay in school, precisely in low-income nations, including India [13, 14].

The caste system is a form of social stratification in which castes are hierarchically organized and separated from each other by rules of ritual purity. Caste is a closed system of stratification, which limits inter-caste interaction and influences a person’s social status based on the caste in which they were born [15]. The caste system has played a significant role in shaping the occupations, roles, and values of Indian society [16]. Caste is also crucial in determining access to social and economic resources [17, 18]. The castes are usually divided into four categories: Scheduled Castes (SC), Scheduled Tribes (ST), Other Backward Classes (OBC), and General castes. The SCs refer to groups of historically disadvantaged people seen as the lowest in the Indian caste structure. Indian constitution defines SC as “such castes, races or tribes or parts of or groups within such castes, races or tribes as are deemed under Article 341 to be SC for this constitution”. Similarly, STs are defined as “such tribes or tribal communities or parts of or groups within such tribes or tribal communities as are deemed under Article 342 to be ST for this constitution”. The OBC is a collective term for castes that are thought to be socially and educationally disadvantaged. The OBC groups are listed in Article 340 as backward classes [19]. The population that does not fall under the SC, ST, and OBC categories is the General/forward caste. In India, caste affects health [20, 21] and is often explained through genetics, early environment, and opportunities due to social mobility [22]. In most socioeconomic development and health indicators, the backward castes/tribes lag significantly behind the General castes. The socially backward castes have inadequate access to healthcare [17, 23], poor maternal health [24, 25], higher fertility [26], and are at greater risk of neonatal and infant mortality [27]. Among the castes, STs, followed by SCs, are the most socioeconomically deprived, with low literacy rates, poor economic conditions, and limited access to healthcare [28, 29]. Along with the STs, the SCs also have high fertility rates and higher newborn and under-five mortality rates, greater than the national average [30]. Moreover, adolescent girls belonging to lower castes are at a heightened risk of early marriage and pregnancy [31–33].

Over the year, the Government of India has launched several affirmative policy measures, such as reserving seats in education and employment, including reservation of seats in parliament and state legislative assemblies for the socioeconomic development of socially backward groups/castes. The Ministry of Health & Family Welfare also sets aside a sizeable budget under SC and Tribal Sub-Plan. Accredited Social Health Activists (ASHA), Auxiliary Nurses and Midwives (ANM), and Staff nurses have been instructed to give special attention to the health of the vulnerable sections, i.e., SCs and STs [34]. These measures are expected to reduce inter-caste inequity in health outcomes and improve the backward castes’ health and welfare [35].

Literature reveals inter-caste disparity in fertility and health outcomes, with women from the socially weaker castes/tribes being the most vulnerable groups. It is also evident that early childbearing can result from several sociocultural, economic, environmental, individual, and health service-related factors, including the outreach of policies and programs aimed at preventing it. Nonetheless, there is a dearth of empirical evidence about the impact of caste on early childbearing, more specifically, regarding the trajectory of inter-caste disparities in early childbearing. Understanding the significance of caste as a predictor of early childbearing would help assess the contributory role of caste-specific affirmative policies/programs aimed at the welfare of the backward castes. Moreover, this will reflect the outreach of family planning programs/policies among the socially backward groups over the year. Against this backdrop, this study assesses the association between caste and early childbearing in India over the last three decades.

## Methods

### Data

**Fig. 1:**
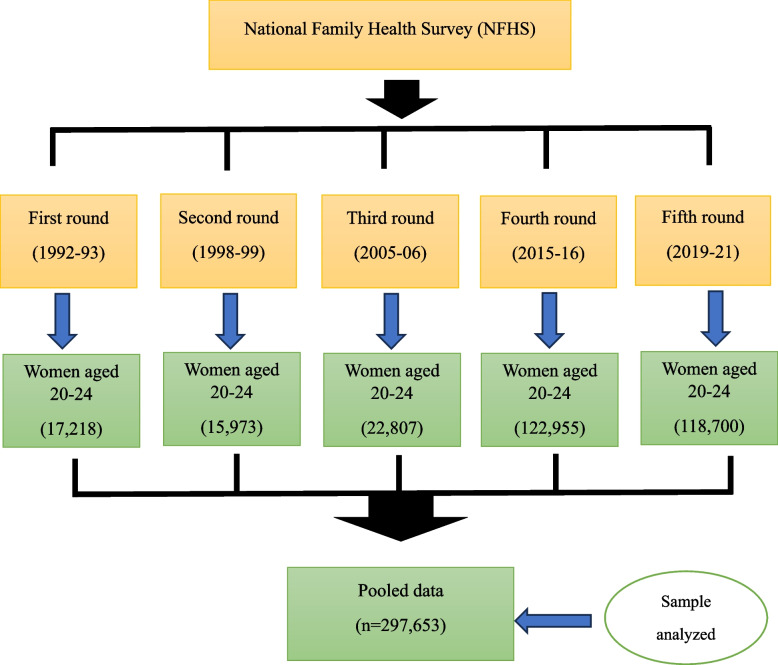
Flowchart of sample selection

The study used data from all five rounds of the National Family Health Survey (NFHS) conducted during the last three decades, i.e., NFHS-1 (1992-93), NFHS-2 (1998-99), NFHS-3 (2005-06), NFHS-4 (2015-16) and NFHS-5 (2019-21). The NFHS is a nationally representative survey that provides data on numerous health and empowerment indicators, including fertility. The NFHSs employ a two-stage stratified sampling technique to select respondents, i.e., women of reproductive age. Only those who voluntarily consented to be interviewed were interviewed. Trained research investigators gathered the data using computer-assisted personal interviewing (CAPI). The report details the survey protocol, sampling, data collection tools, and quality control measures [6]. In this study, all the women aged 20–24 [NFHS-1 (*n* = 17,218), NFHS-2 (*n* = 15,973), NFHS-3 (*n* = 22,807), NFHS-4 (*n* = 122,955) and NFHS-5 (*n* = 118,700)] were considered to create a pooled data set (*n* = 297,653) for analysis (Fig. 1). We considered the women aged 20–24 for analysis to avoid any possible overlapping of the same cohort of women/women with similar characteristics in different survey rounds.

### Outcome variable

The outcome variable of this study was ‘early childbearing,’ defined as percentages of women who started childbearing (either had given birth or were pregnant) before 19 years of age. Past studies considered different ages of the women to describe early childbearing, such as below 20 years [36] and 18 years [37]. However, this study considered below 19 to capture all adolescent pregnancies and childbirths. Enough evidence exists that signifies the adverse outcomes of pregnancies/births to mothers below 19 years [38, 39]. World Health Organization (WHO) also views that adolescent (10–19 years) pregnancy results in no biological, mental, or social maturation process and adversely affects maternal and fetal outcomes due to biological immaturity, inadequate antenatal care, malnutrition, unhealthy habits, stress, depression, and anxiety [40].

### Predictor variables

The key predictor variable used in the analysis was ‘caste,’ categorized as SC, ST, OBC, and General caste. The NFHS-1 did not have a separate OBC category; OBC data was presented as a part of the General caste category. Additionally, selected socioeconomic and demographic factors such as years of schooling (no, less than ten years, ten or more years), mass media exposure (yes, no), religion (Hindu, Muslim, and others), wealth quintile (poorest, poorer, middle, richer, richest), place of residence (urban, rural), region (south, north, central, east, northeast, and west) and time (NFHS-1, NFHS-2, NFHS-3, NFHS-4 and NFHS-5) were included in the analysis to assess the adjusted effect of caste on early childbearing.

### Statistical analysis

Bivariate analysis and Chi-square test were performed first to check the association between the predictor and outcome variables. Binary logistic regression was further conducted to check the adjusted effects of caste on early childbearing. The variables for the regression analysis were finalized after checking the multicollinearity among the predictor variables through the VIF^1^ method [41]. In the analyses, weights were used to restore the sample’s representativeness. “A design weight is calculated to account for the overall selection probability of each household in the survey. The design weight is adjusted for household non-response and for individual non-response to obtain the sampling weights for households, for women, and for men, respectively. For national weights, the sampling weights are normalized to give a total number of weighted cases that equals the total number of unweighted cases at the national level. Normalization is done by multiplying the sampling weight by the estimated sampling fraction, calculated on the national level for national weights” [42]. The analyses were performed using Stata (version 17) with a significance level of 5%. ArcMap (v10.8) presented the most recent caste-wise prevalence of early childbearing among states and Union Territories (UTs).

## Results

### Trend of early childbearing by caste

**Fig. 2:**
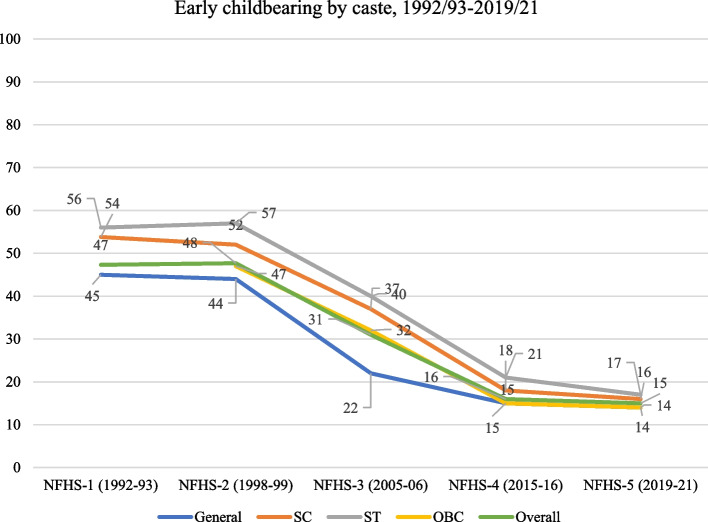
Trends of early childbearing among women aged 20–24 by caste, India,1992-93 to 2019-21

**Fig. 3:**
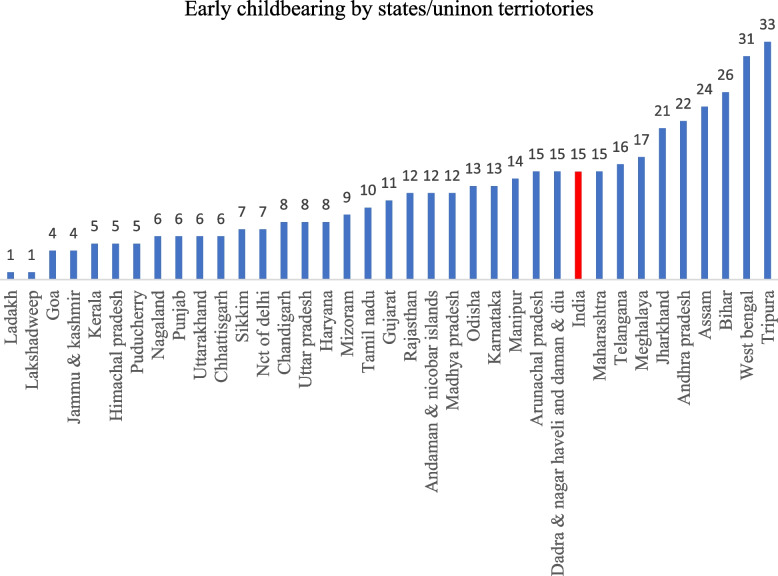
Early childbearing among women aged 20–24 by states/union territories, India, 2019-21

**Fig. 4:**
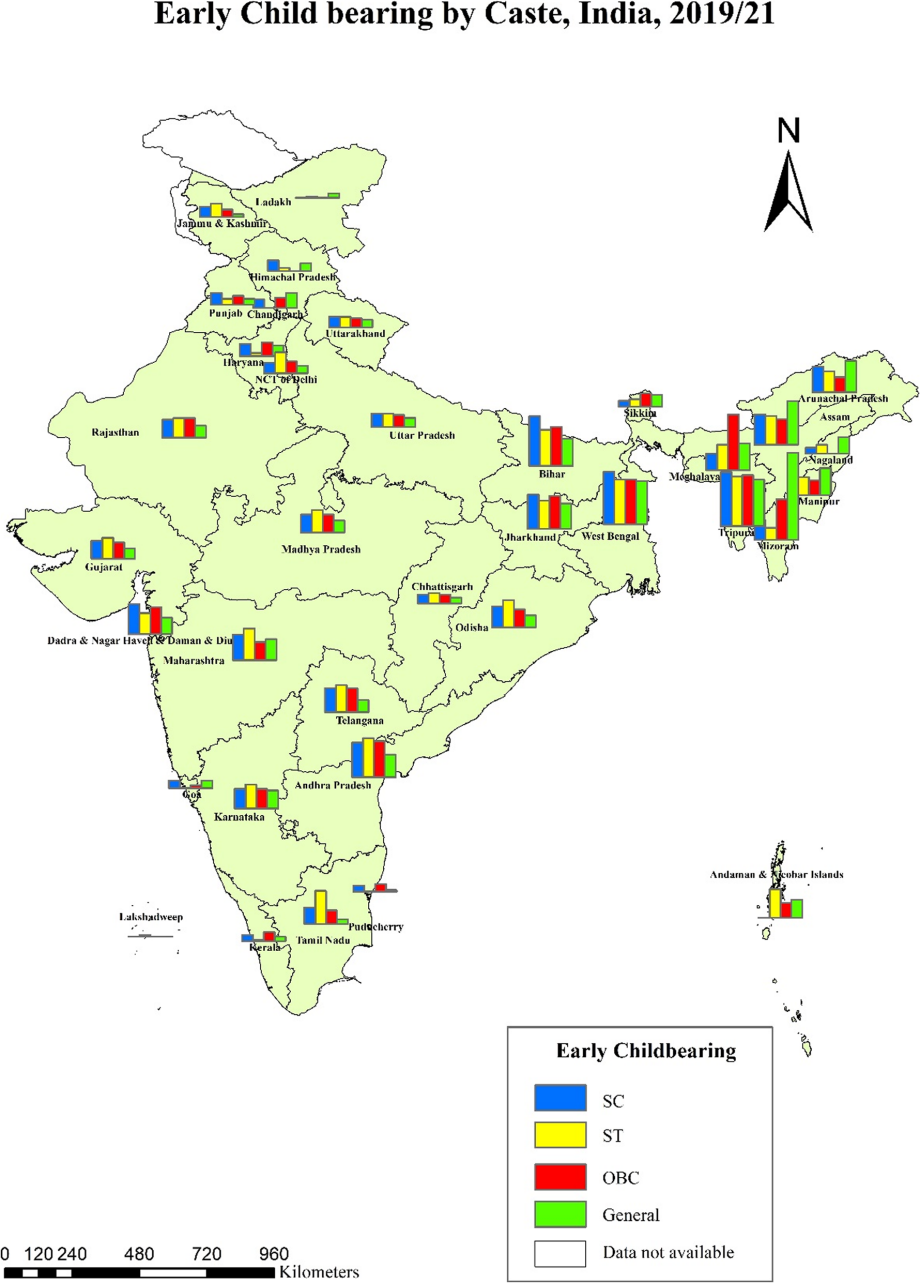
Early childbearing among women aged 20–24 by caste, India, 2019/21

The percentage of women with early childbearing reduced gradually over the last three decades- from 47% in 1992-93 to 15% in 2019-21 (Fig. 2). The steepest decline occurred between 2005 and 06 and 2015-16 (48% points), followed by 1998-99 to 2005-06 (35% points). In the last three decades, there was a steady decline in early childbearing across the castes − 70% points for both SCs and STs and 69% points for the General castes. Nevertheless, early childbearing continued to be high among socially backward groups, i.e., SCs (16%) and STs (17%), compared to the General caste (14%). In 2019-21, among states and UTs, Tripura had the highest prevalence of early childbearing (33%), followed by West Bengal (31%) and Bihar (26%) (Fig. 3). Caste-wise, Tripura had the highest percentage of early childbearing for SCs and STs, Meghalaya had the highest percentage for OBCs, and Mizoram had the highest percentage for General caste (Fig. 4).

### Socioeconomic differential in early childbearing

Table 1 presents the socioeconomic differential in early childbearing from 1992 to 93 to 2019-21. One-fifth of SC and ST women began childbearing early. The corresponding figures for the OBCs and General caste was 16% and 19%, respectively. Early childbearing prevalence declined with increasing education, exposure to mass media, and increasing women’s economic condition across all the survey rounds. Moreover, early childbearing prevalence was lower among non-Hindus/Muslims and women from urban areas. Early childbearing was most common among women from the eastern region (26%), while it was least common among women from the northern region (13%).

**Table 1:** Early childbearing by socioeconomic and demographic characteristics of women aged 20–24, India (Pooled data of 1992-93 to 2019-21)

Background characteristics	Started childbearing below 19 years		Chi-square *p*-value
	Percentage (%)	Total women	
**Caste**^**a**^			
General	19.4	82,914	0.000
SC	19.5	55,636	
ST	19.7	51,130	
OBC	16.1	1,06,449	
Don’t know	27.9	1,401	
**Years of schooling**^**a**^			0.000
No schooling	40.0	50,370	
< 10 years	24.7	1,01,390	
10 or more years	6.5	1,45,884	
**Mass media exposure**^**a**^			0.000
No	31.2	62,806	
Yes	14.9	2,34,825	
**Religion**^**a**^			0.000
Hindu	18.5	2,22,652	
Muslim	20.5	42,136	
Others	14.6	32,846	
**Wealth index**			0.000
Poorest	27.4	52,727	
Poorer	22.6	62,513	
Middle	18.4	63,780	
Richer	14.4	62,493	
Richest	9.3	56,140	
**Place of residence**			0.000
Urban	13.3	82,290	
Rural	20.3	2,15,363	
**Region**			0.000
South	19.4	40,347	
North	12.8	61,870	
Central	15.5	76,534	
East	26.1	52,065	
North-east	21.1	39,583	
West	18.4	27,254	
**Time**			0.000
NFHS-1 (1992-93)	43.7	17,218	
NFHS-2 (1998-99)	44.5	15,973	
NFHS-3 (2005-06)	23.9	22,807	
NFHS-4 (2015-16)	15.0	1,22,955	
NFHS-5 (2019-21)	13.5	1,18,700	
**Total**	**18.3**	**297,653**	

^a^May not match to total women due to missing cases

### Early childbearing by age and caste

**Fig. 5:**
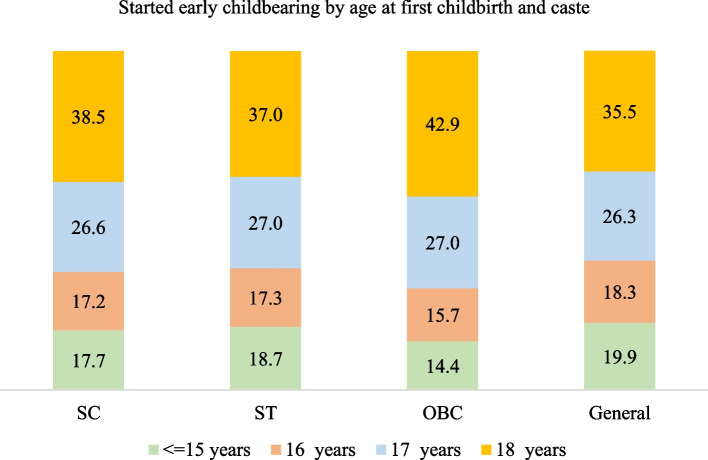
Early childbearing among women aged 20–24 by age at first child birth and caste, India, 1992-93 to 2019-21

Figure 5 presents early childbearing by age at first childbirth and caste. Irrespective of caste, a majority of the women aged 20–24 who started early childbearing had their first birth at age 18. Of the General caste women with early childbearing, 20% had their first birth by 15 years of age. The corresponding figures were 19% for STs, 18% for SCs and 14% for OBCs.

### Determinants of early childbearing

Table 2, based on the pooled data, presents the logistic regression results for the association of caste with early childbearing. Adjusting the effects of socio-demographic and economic characteristics, SC women had significantly higher odds of early childbearing (OR = 1.07, CI = 1.04–1.11) than those from the General category. Compared to NFHS-1 (1992-93), the odds of early childbearing increased by 15% (OR = 1.15, CI = 1.09–1.20) in NFHS-2 (1998-99), and thereafter declined by 42% (OR = 0.58, CI = 0.55–0.60) in NFHS-3 (2005-06), 64% (OR = 0.36, CI = 0.35–0.38) in NFHS-4 (2015-16), and NFHS-5 (2019-21).

**Table 2:** Adjusted Odds Ratio (AOR) of determinants of early childbearing of women aged 20–24, India, 1992-93 to 2019-21

Background characteristics	AOR	95% CI
**Caste**		
General ®	[1.00–1.00]	
SC	1.07***	[1.04–1.11]
ST	0.95	[0.92–0.99]
OBC	1.01	[0.98–1.04]
Don’t know	1.35***	[1.19–1.53]
**Years of schooling**		
No schooling ®	1	[1.00–1.00]
< 10 years	0.65***	[0.63–0.66]
10 or more years	0.17***	[0.17–0.18]
**Mass media exposure**		
No ®	1	[1.00–1.00]
Yes	0.88***	[0.86–0.90]
**Religion**		
Hindu ®	1	[1.00–1.00]
Muslim	1.06***	[1.03–1.10]
Others	0.73***	[0.70–0.76]
**Wealth index**		
Poorest ®	1	[1.00–1.00]
Poorer	1	[0.98–1.04]
Middle	0.92***	[0.89–0.95]
Richer	0.80***	[0.77–0.83]
Richest	0.62***	[0.60–0.65]
**Place of residence**		
Urban ®	1	[1.00–1.00]
Rural	1.13***	[1.10–1.16]
**Region**		
South ®	1	[1.00–1.00]
North	0.52***	[0.50–0.54]
Central	0.54***	[0.52–0.56]
East	0.96*	[0.93-1.00]
North-east	0.99	[0.94–1.02]
West	0.77***	[0.74–0.81]
**Time**		
NFHS-1 (1992-93) ®	1	[1.00–1.00]
NFHS-2 (1998-99)	1.15***	[1.09–1.20]
NFHS-3 (2005-06)	0.58***	[0.55–0.60]
NFHS-4 (2015-16)	0.36***	[0.35–0.38]
NFHS-5 (2019-21)	0.36***	[0.35–0.38]

* *p* < 0.05, *** *p* < 0.001

® Reference category, *CI* Confidence interval

## Discussion

The study found that many women continue to have early childbearing despite a considerable reduction over the last three decades. Inter-caste disparities in early childbearing persist, albeit with a narrowing gap, with the SC women remaining the most vulnerable group. We found many women are bearing children early, which conforms to earlier studies that revealed that early childbearing rates are consistently high [33, 43, 44]. This study found that SC women are more likely to bear children early than their General caste counterparts. Accounting for other variables, a past study revealed that the SC girls were, on average, 10% more likely to give birth early than those from the other castes [43]. Several other studies also found that lower-caste adolescent girls are at higher risk of early marriage and pregnancy [31, 32]. The study found that ST women are less vulnerable to early childbearing than SC women, although they exhibit equal socio-economic backwardness. This may be due to the better status of ST women owing to the distinctiveness of tribal cultures that leads to less discrimination of ST women than those from SCs, in addition to higher age at marriage and autonomy in fertility preference [45].

A relatively higher percentage of SC women continue to be illiterate than those from the General caste. For example, in 1992-93, 25% of SC women were literate, which increased to 90% in 2019-21, an annual increase of 2.2%. The corresponding figure for the General caste was 1.6% (47–94%) [6]. Enough literature reveals that inadequate education enhances the chance of early pregnancy, often through early marriage, inadequate awareness of contraception and its use, and low healthcare autonomy [43]. In 2019-21, more than half (51%) of the SC households had a Below Poverty Line (BPL) card compared with 33% of the General caste households [6]. A recent estimate also reveals that compared to the advantaged groups, the multidimensional poverty level is two times higher among the SCs [46]. It is proven that poverty often contributes to early marriage [47], which further contributes to inadequate use of contraception and, subsequently, early pregnancy [48]. Moreover, more SC women continue to marry below 18 than their counterparts from the General caste. For example, in 1992-93, 69% of SC women got married by 18, which decreased to 39% in 2019-21, a decrease of 1.03% annually. The corresponding figure for the General caste was 0.8% (59%- 37%) [6]. Social norms determine early marriage and pregnancy [49]. Marriage below 18 is significantly associated with women’s increased risk for no contraceptive use before first childbirth, high fertility, history of rapid repeat childbirth, and multiple unwanted pregnancies [50]. Early marriage again limits contraceptive use [51], thus resulting in early pregnancy.

The present study empirically proves that the inter-caste differentials in early childbearing are reducing, which is a positive social change. Narrowing inter-caste disparity in early childbearing also suggests the outreach of affirmative policies and programs aimed at the socio-economic development of the backward caste/tribes. A recent study highlights the contributory role of the implementation of social programs for women and girls’ school enrollment in the rise in age at marriage among the SCs, a significant predictor of early childbearing [52]. At the same time, the overall higher prevalence of early childbearing indicates the existing norms on marriage age and pregnancy immediately after marriage in Indian society. A past study revealed that gender norms significantly influence early fertility desires, but interventions frequently ignore this factor, especially for marginalized groups [53].

Early childbearing harms young women’s health and development and contributes to neonatal morbidity, mortality, and child development, more so among socially disadvantaged groups. Delaying the marriage age has been proven to delay the birth of the first child for young women [54], thus improving the health of younger women by reducing complications during pregnancy and childbirth. A past study suggests five promising interventions to address child marriage and early childbirth for girls, such as (a) an increasing opportunity for girl’s friendly higher education, (b) financial assistance to reduce the financial burden of the family regarding marriage expenses, (c) improved access to economic opportunities, (d) proper awareness regarding gender and social norms, and (e) special assistance for newlyweds, including access to sexual and reproductive health services to help young married couples delay getting children [55]. An intervention-based study found that strategies such as community-level youth information centers and exposure to mass media significantly reduce early marriage and early pregnancy among adolescents [32]. Another study assessing child marriage prevention measures in the country also suggests interventions aiming at increased attendance at school and changing social attitudes to child marriage are two effective ways that benefit society and the economy [56]. It is worth mentioning that several Indian states have programs that provide monetary assistance to financially strapped families for the marriage expenses of daughters. For example- *Rupashree Prakalpa* in West Bengal ensures a one-time incentive of 25,000 Indian Rupees (INR) to low-income families when adult daughters get married [57]. In Uttar Pradesh, poor families from minority communities are entitled to a one-time amount of INR 51,000 for a daughter’s marriage under the *UP Shadi Vivah Anudan Yojana* [58]. In Odisha, girls from the Particularly Vulnerable Tribal Groups (PVTG) are given a one-time cash incentive of INR 20,000 for marrying after 18 years of age [59].

The study’s strengths are that the findings are based on large-scale, nationally representative samples chosen using a robust sampling design. The study presents the level and trajectory of inter-caste disparity in early childbearing for the last three decades; thus, the results are imperative and relevant. The results contribute to the existing scanty evidence on the role of caste in fertility preference, which may also apply to similar settings in other South-Asian countries like Nepal, Pakistan, and Sri Lanka. However, the cross-sectional design of NFHS limits any causal inference drawn from this analysis. In the NFHS-1, OBCs were included in the General caste category; thus, care must be taken while comparing the caste groups over time. There is also a possibility of an intersectional vulnerability affecting early childbearing among caste groups in India. Additionally, social norms and contextual factors influence early childbearing, which this analysis could not include due to a lack of information.

## Conclusion

Caste continues to be a significant predictor of early childbearing, and SC women are the most susceptible, though there has been a substantial reduction in inter-caste disparity in early childbearing in the last three decades. Early childbearing among the socially backward groups is often influenced by their early marriage, low/no contraceptive use, and inadequate healthcare autonomy; those again determined by their inadequate education and poor economic condition; thus, ongoing programs and policies aimed at educational and economic empowerment of the socially weaker groups should be strengthened. The result further suggests an emphasis on preventing adolescent marriages and expanding contraceptive use among young SC women to reduce early childbearing. Efforts to prevent early childbearing will accelerate the achievement of the Sustainable Development Goals (SDGs)-especially those related to health, poverty, nutrition, education, and general wellbeing, in addition to protecting women’s reproductive rights.

## Data Availability

The datasets generated and/or analyzed during the current study are available in the Demographic and Health Surveys Repository through individual registration [https://www.dhsprogram.com/data/new-user-registration.cfm].

## Abbreviations

ST: Scheduled Tribe
SC: Scheduled Caste
OBC: Other Backward Classes
NFHS: National Family Health Survey
CAPI: Computer-Assisted Personal Interviewing
OR: Odds Ratio
CI: Confidential Interval
INR: Indian Rupee
PVTG: Particularly Vulnerable Tribal Groups
VIF: Variance Inflation Factor
UT: Union Territory
ASHA: Accredited Social Health Activist
ANM: Auxiliary Nurse and Midwife
WHO: World Health Organization

## Footnotes

1 Variance inflation factor (VIF) is one of the tests for checking the multicollinearity among the predictor variables. “The variance of regression coefficients is proportional to $\frac{1}{(1-{R}^{2})}$, which is called the variance inflation factor. Considering the range of R2 (0 ≤ R2 ≤ 1), R2 = 0 (complete absence of multicollinearity) minimizes the variance of the regression coefficient of interest, while R2 = 1 (exact multicollinearity) makes this variance infinite. The reciprocal of the variance inflation factor (1 − R2) is known as the tolerance. If the variance inflation factor and tolerance are greater than 5 to 10 and lower than 0.1 to 0.2, respectively (R2 = 0.8 to 0.9), multicollinearity exists” [41].
